# The misinterpretation of structure effects of the LMDI and an alternative index decomposition

**DOI:** 10.1016/j.mex.2022.101698

**Published:** 2022-04-12

**Authors:** Nicolas Roux, Barbara Plank

**Affiliations:** Institute of Social Ecology (SEC), Department of Economics and Social Sciences, University of Natural Resources and Life Sciences, Vienna, Schottenfeldgasse 29, Vienna 1190, Austria

**Keywords:** Index decomposition analysis (IDA), Logarithmic mean divisia index (LMDI), Structure effects, Mix effects, Marshall-edgeworth decomposition, LMDI, Logarthmic Mean Divisia Index, IDA, Index Decomposition Analysis, MESE, Marshall-Edgeworth with Structure Effects

## Abstract

Changing the structure of the economy is often considered an option to reduce environmental impacts – for example, by changing the mix of sectors in the economy, the energy mix of production, or the mix of origin countries for imported products. To study the effect of such structure (or mix) effects, researchers often use index decomposition analysis (IDA). This study uses experimental data to show that most existing IDA methods, especially the widely used LMDI (logarithmic mean divisia index), yield results that are difficult to understand and easily misinterpreted. We use formal proof to demonstrate that:

• The LMDI interpretation problem is due to the use of shares to describe the considered mix.
• We developed an alternative method, the Marshall-Edgeworth with Structure Effects (MESE).
• The MESE defines structure effects by comparing each observation to a hypothetical average, which better reflects the common understanding of structure effects.

We compared empirical data on the LMDI and the MESE, analysing the effect of the changing sector mix on energy use in the USA from 1995 to 2016, and found that results from the two tools differed significantly. We therefore recommend using the MESE when structure effects are included in IDA.

## Graphical abstract

**Figure:**
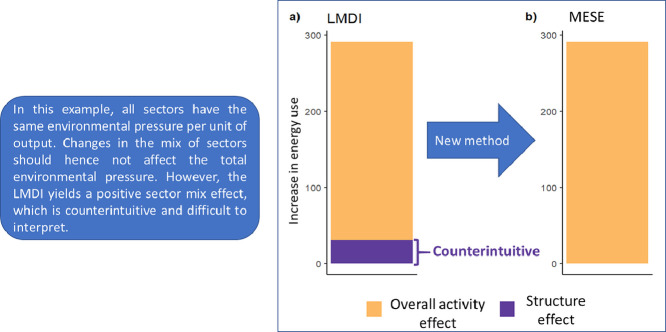

*Specifications table*

**Table:** 

Subject Area:	Environmental Science
More specific subject area:	*Index decomposition analysis*
Method name:	Marshall-Edgeworth with Structure Effects (MESE)
Name and reference of original method:	Logarithmic Mean Divisia Index (LMDI) Ang, B.W. (2005) The LMDI approach to decomposition analysis: a practical guide, *Energy Policy*, 33(7), pp. 867–871. doi:10.1016/j.enpol.2003.10.010.
Resource availability:	An R package to implement this method can be found on GitHub (https://github.com/ncrx5/MEME.decomposition.git)

* Method details

## Introduction

Changes in the structure of the economy significantly determine countries’ impacts on the environment. Hence, researchers have attempted to quantify these structure (or mix^1^) effects on environmental impacts. Common structure effects include changes in the sector mix of the economy [3,8,16], the fuel mix of energy production [8,13], the product mix of typical consumption baskets [1,15], and the mix of countries where consumed commodities are produced and imported [14,17,20]. Although structure effects tend to be a little smaller than other drivers of environmental impacts such as overall economic activity or technology, studies have shown that structure effects can be significant [8,10,12,24].

For example, some economies have been specialising in energy-efficient sectors in recent decades, while others have increased their production in energy-intensive sectors, affecting energy use and related greenhouse gas emissions and air quality [10]. Changes in the mix of goods produced by the US economy between 1997 and 2002 are said to have decreased aggregate energy demand by 19.1% [22]. The restructuring of countries´ economies towards environmentally efficient sectors is usually a strategy to reduce energy use and decarbonise economies. It is therefore vital to consistently quantify the impact on the environment of these changes in economic structure in relation to other drivers such as economic growth and technology.

The drivers of environmental impacts are often analysed using index decomposition analysis (IDA) [2,3,11,18]. IDA attempts to explain the change in an aggregate environmental variable (e.g., global energy consumption, CO_2_ emissions or land use) by dividing it into changes in various underlying drivers. IDA often reflects the idea that changes in environmental impacts are driven by changes in economic activity, in environmental impact per unit of output, and in the structure of the economy [3,8,10,12,16,19,20,24].

There are various methods for performing an IDA. These have been extensively compared [3,4,11]. There are two broad IDA methods: the Laspeyres index and the Divisia index. Both have benefits and drawbacks. First, conventional Laspeyres indices leave potentially large residuals that can hamper the interpretation of results. Various corrections have been applied to eliminate residuals, leading to refined Laspeyres and modified Fischer ideal indices [3,6]. Nonetheless, the latter have been criticised for their formula becoming disproportionally complex when the number of considered drivers increases. Divisia indices include the arithmetic mean divisia index (AMDI) and the log mean divisia index (LMDI). Following the recommendations of Ang [2,3], the LMDI has become the most commonly used decomposition approach. The LMDI yields a perfect decomposition (i.e., it leaves no residual term), while its formula for each driver is concise and independent of the number of drivers. Consequently, the LMDI has been widely applied by researchers, public institutions and statistical offices such as Eurostat for deriving policy recommendations and performing environmental impact assessments [9,18].

The common understanding of structure effects is that when the economy shifts towards products, sectors or producing countries associated with comparatively high environmental impacts per unit of product, the total environmental impact deteriorates. However, nearly all existing IDA methods, including the LMDI, define structure effects as changes in each sector, fuel, or origin region's shares in the total economy. Changes in these shares are treated as a driver of environmental impact in the same manner as overall activity or energy intensity. This approach offers simplicity and mathematical elegance. However, we argue that treating shares similarly to other drivers is problematic because it does not semantically correspond to the common understanding of structure effects. Hence, we suggest that approaches such as the LMDI, which use shares to measure structure effects, generate results that can easily be misinterpreted. In particular, structure effects calculated with the LMDI cannot be interpreted as the sole effect of changes between energy-efficient and inefficient sectors. We therefore suggest an alternative method that better reflects the common interpretation of structure effects and was first used in Roux et al. [20].

Section 1 "*Introduction*" introduces the interpretation problem of the LMDI based on an experimental example. Section 2 formally demonstrates that the interpretation problem of the LMDI is linked to the use of shares to depict structure effects. Section 3 describes the MESE, an alternative method whose results are closer to common interpretations. Section 4 compares the LMDI with the MESE, using an empirical example (energy carrier use in the USA between 1995 and 2016). Section 5 discusses some desirable properties of the new method as well as some drawbacks. Finally, we provide some thoughts on the relevance of this method for policy analysis.

### Experimental example illustrating the difficulty of interpreting structure effects of the LMDI

To make our argument more tangible, assume we want to decompose the drivers of an environmental pressure *E* (e.g., energy consumption). A basic decomposition of E would be:

(1.1) E=Y×I

where Y is the total economic output, and I is the energy intensity (energy consumption per unit of output). Many applications include other factors such as population or energy requirement, which we omitted here for simplicity. For an application with three initial factors, refer to the appendix.

Let us assume that the output can be separated into different sectors of different energy intensity. We therefore want to depict the effect of changes in the sector mix.^2^ In this context, the sector mix effect is commonly understood as the effect on energy consumption when specialising in sectors associated with a comparatively high energy use per product unit. For example, if a country originally specialised in sectors associated with low energy use per unit of output and starts producing goods with higher energy use per unit of output, it should worsen (i.e., increase) the sector mix effect. Reciprocally, the sector mix effect decreases when that country increases its production in sectors associated with a lower energy use per unit of output.

In conventional approaches, structure effects are usually incorporated into Eq. (1.1) as follows:

(1.2) $$E=\sum_{i}Y\times S_{i}\times I_{i}$$

where the subscript *i* stands for the different sectors. $S_{i}=\frac{Y_{i}}{Y}$ is the share of sector *i* in the total economy.

Eq. (1.2) shows that the shares $S_{i}$ are treated like the other drivers Y and $I_{i}$.

In the LMDI, the contribution attributed to any of these drivers (X) is calculated as follows:

$$\Delta E_{X}=\sum_{i}L\left(E_{i}^{t},\;E_{i}^{0}\right)\times ln\left(\frac{X_{i}^{t}}{X_{i}^{0}}\right)$$

where

(1.3) $$L\left(E_{i}^{t},\;E_{i}^{0}\right)=\frac{E_{i}^{t}-E_{i}^{0}}{\text{ln}(E_{i}^{t}/E_{i}^{0})}$$

The difficulty of interpreting the results of the LMDI when analysing structure effects holds for any value of the other drivers. However, it is most evident in the situation where the energy intensity (energy use per unit of output) is equal in all sectors. Hence, we will give an example where all sectors are attributed the same intensity. Logically, changes in the sector mix should not influence total energy consumption, because it would not matter in which sector the products are produced (i.e., all sectors are equally energy-intensive). In other words, the logical outcome in this example would be that the sector mix effect should be nil for any change in production. However, we show that if changes in different sectors do not cancel each other out and sectors do not change in the same proportion, the LMDI allocates a large share of this change to the structure effect, which is counterintuitive, given that all sectors produce with the same intensity.

**Fig. 1:**
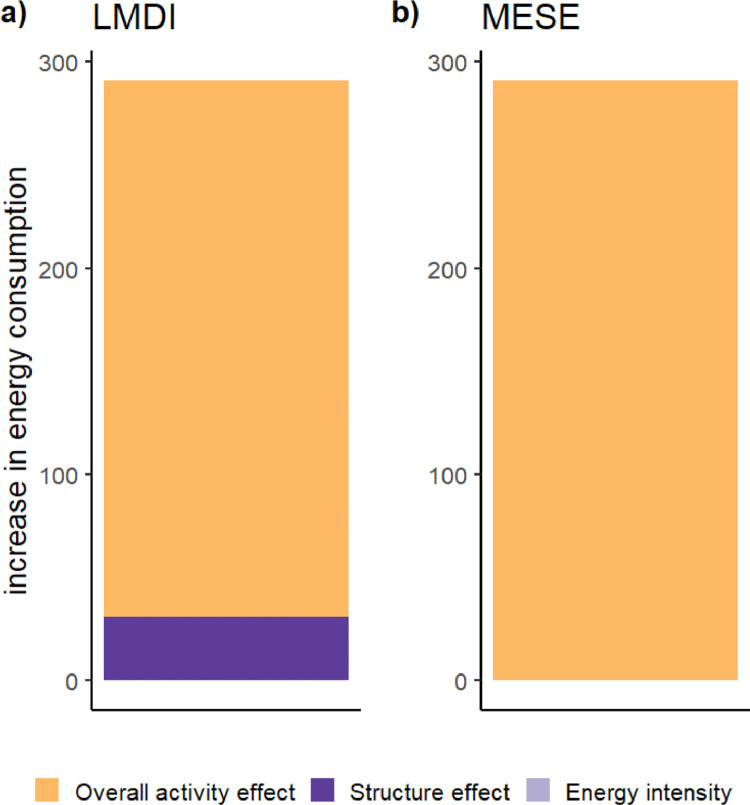
Results of the illustrative example using (a) the LMDI and b) the MESE decomposition. Although all sectors have the same energy intensity, the LMDI allocates 11% of the change in energy consumption to the sector mix, which contradicts the intended semantics of a structure effect. This problem disappears using the MESE.

In this simple example (Table 1), sectors *a, b* and *c* have the same intensity. As we have argued, the sector mix effect should be zero, according to semantic reasoning. Sector *a* undergoes an increase in a different proportion to sectors b and c. Hence, suppose sector *a*’s output is multiplied by 90 while the output of sectors *b* and *c* is multiplied by 5, and everything else is kept equal. In this simple case, the LMDI allocates 11% of this increase to the sector mix (Fig. 1), which is a result that contradicts the intended semantics of a structure effect. As the sector should not matter in this case, every increase should be allocated to the effect of overall economic activity. This problem disappears using the MESE.

**Table 1:** Example illustrating the problem of interpreting the LMDI.

sector	Year	Energy consumption	Output	Overall activity	Share of sector	Energy intensity	total energy consumption
a	0	3	1	3	0.33	3	9
b	0	3	1		0.33	3	
c	0	3	1		0.33	3	
							
a	t	270	90	100	0.9	3	300
b	t	15	5		0.05	3	
c	t	15	5		0.05	3

### The difficulty in interpreting the structure effects of the LMDI is due to the use of shares: formal proof

In this section, we demonstrate that the difficulty in interpreting the structure effects in the LMDI is due to the use of shares to define the structure of the economy. Our proof is composed of three steps. Claims 1 and 2 describe two extreme cases where the LMDI is not subject to the problem of misinterpretation. Claim 3 reveals the problem of the LMDI for all situations other than 1 or 2.

Exception 1 **The perfect switch – no misinterpretation**

When one country completely replaces the production from certain sectors with production from other sectors, the structure effect calculated by the LMDI is a sum of the changes in each sector's output, weighted by their respective energy intensity. This is coherent with the interpretation of the structure effect, because when one country replaces production from an energy efficient sector with production from an energy intensive sector, the structure effect will increase. Furthermore, when the switch is between sectors with the same energy intensity, their respective contribution to the structure effect will cancel each other out as expected. Therefore, in this situation, the LMDI does not suffer from any misinterpretation.

Alt-text: Unlabelled box

Proof of Claim 1:

Assume one country completely switches its production between several sectors without increasing its total national output. In other words, taking 0 as the base year and t as the comparison year: $\sum_{i}\Delta Y_{i}=0$

All other drivers (except the shares) are assumed to be constant: calling $A_{i}=Y\times I_{i}$ gives $A_{i}^{t}=A_{i}^{0}$. Rewriting Eq. (1.3), the effect attributed to the shares (the structure effect) is:

$$\Delta E_{S,i}=\frac{E_{i}^{t}-E_{i}^{0}}{ln\left(\frac{A_{i}^{t}S_{i}^{t}}{A_{i}^{0}S_{i}^{0}}\right)}\times ln\left(\frac{S_{i}^{t}}{S_{i}^{0}}\right)=\frac{E_{i}^{t}-E_{i}^{0}}{ln\left(\frac{S_{i}^{t}}{S_{i}^{0}}\right)}\times ln\left(\frac{S_{i}^{t}}{S_{i}^{0}}\right)=\Delta E_{i}$$

Calling $Y_{i}=Y\times S_{i}$ the size of sector *i*, the following can be written:

$$\Delta E_{S,i}=I_{i}\times \Delta Y_{i}$$

Finally:

$$\Delta E_{S}=\sum_{i}I_{i}\times \Delta Y_{i}$$

with $\sum_{i}\Delta Y_{i}=0$ which proves our Claim 1.

Note that when all sectors are associated with the same intensity ($\forall i,\;I_{i}=I$), the sector mix effect will be null as expected. However, Claim 1 does not hold when one of the other drivers changes over time ($A^{t}\neq A^{0})$, or when the switch is not perfect ($\sum_{i}\Delta Y_{i}\neq 0)$. An illustrative example of exception 1 can be found in appendix A1.

Exception 2 **Proportional sectoral change – no misinterpretation**

If production changes in the same proportion for all sectors, the shares remain constant for each sector, so that the structure effect is null as expected. The entire change is correctly allocated to the overall activity effect, and the LMDI decomposition is not subject to misinterpretation.

Alt-text: Unlabelled box

Proof of Claim 2:

If production changes in the same proportion for all sectors, the result is:

$$\forall i,\;Y_{i}^{t}=\alpha \times Y_{i}^{0}$$

Therefore,

$$S_{i}^{t}=\frac{\alpha \times Y_{i}^{0}}{\alpha \times Y^{0}}=S_{i}^{0}$$

$$\begin{array}{ccc} & & \frac{S_{i}^{t}}{S_{i}^{0}}=1\\ & & ln\left(\frac{S_{i}^{t}}{S_{i}^{0}}\right)=0 \end{array}$$

Replacing in Eq. (1.3),

$$\Delta E_{S}=0$$

This proves Claim 2.

An illustrative example of exception 2 can be found in appendix A1.

Claim 3 **Non-proportional sectoral change – misinterpretation in the LMDI**

If the switch is not perfect, and the change in production for at least one sector is not in the same proportion as the change in another sector, one part of the effect of overall activity is allocated to the structure effect, which makes it difficult to interpret.

Alt-text: Unlabelled box

Proof of Claim 3:

Assume there is one sector $\tilde{i}$ where size does not change in the same proportion as other sectors (for example, steel production tripled while all other sectors doubled):

$$\exists \tilde{i},:\;Y_{\tilde{i}}^{t}=\beta \times Y_{\tilde{i}}^{0}$$

$$\forall i\neq \tilde{i}:\;Y_{i}^{t}=\alpha \times Y_{i}^{0}$$

andβ≠α

As in the proof of Claim 1, the nominator of the LMDI weights is:

$$E_{i}^{t}-E_{i}^{0}=I_{i}\times \Delta Y_{i}$$

Assuming that intensity is constant, the denominator for $\tilde{i}$ is:

$$ln\left(\frac{E_{\tilde{i}}^{t}}{E_{\tilde{i}}^{0}}\right)=ln\left(\frac{E^{t}\times Y^{t}\times \frac{Y_{\tilde{i}}^{t}}{Y^{t}}\times I_{\tilde{i}}^{t}}{E^{0}\times Y^{0}\times \frac{Y_{\tilde{i}}^{0}}{Y^{0}}\times I_{\tilde{i}}^{0}}\right)=ln\left(\frac{Y_{\tilde{i}}^{t}}{Y_{\tilde{i}}^{0}}\right)=ln\left(\beta \right)$$

Similarly,

$$ln\left(\frac{E_{i}^{t}}{E_{i}^{0}}\right)=ln\left(\alpha \right)$$

Moreover,

$$ln\left(\frac{S_{\tilde{i}}^{t}}{S_{\tilde{i}}^{0}}\right)=ln\left(\frac{\frac{Y_{\tilde{i}}^{t}}{Y^{t}}}{\frac{Y_{\tilde{i}}^{0}}{Y^{0}}}\right)=ln\left(\beta \frac{Y^{0}}{Y^{t}}\right)=ln\left(\beta \right)+ln\left(\frac{Y^{0}}{Y^{t}}\right)$$

and

$$ln\left(\frac{S_{i}^{t}}{S_{i}^{0}}\right)=ln\left(\alpha \right)+ln\left(\frac{Y^{0}}{Y^{t}}\right)$$

Combining the parts, the effect attributed to the change in the share of sector $\tilde{i}$ is:

$$\begin{array}{ccc} \Delta E_{S,\tilde{i}} & = & \frac{I_{\tilde{i}}\times \Delta Y_{\tilde{i}}}{ln\left(\beta \right)}\times \left(ln\left(\beta \right)+ln\left(\frac{Y^{0}}{Y^{t}}\right)\right)\\ & = & I_{\tilde{i}}\times \Delta Y_{\tilde{i}}+\frac{I_{\tilde{i}}\times \Delta Y_{\tilde{i}}}{ln\left(\beta \right)}\times ln\left(\frac{Y^{0}}{Y^{t}}\right) \end{array}$$

Finally, the total structure effect calculated by the LMDI is:

$$\Delta E_{S}=\sum_{i}I_{i}\times \Delta Y_{i}+\frac{I_{\tilde{i}}\times \Delta Y_{\tilde{i}}}{ln\left(\beta \right)}\times ln\left(\frac{Y^{0}}{Y^{t}}\right)+\sum_{i\neq \tilde{i}}\frac{I_{i}\times \Delta Y_{i}}{ln\left(\alpha \right)}\times ln\left(\frac{Y^{0}}{Y^{t}}\right)$$

For the sake of generalization, we define $\alpha_{i}=\frac{Y_{i}^{t}}{Y_{i}^{0}}$ and $\exists \tilde{i},i,$: $\alpha_{\tilde{i}}\neq \alpha_{i}$, we obtain:

$$\Delta E_{S}=\sum_{i}I_{i}\times \Delta Y_{i}+\sum_{i}\frac{I_{i}\times \Delta Y_{i}}{ln\left(\alpha_{i}\right)}\times ln\left(\frac{Y^{0}}{Y^{t}}\right)$$

$\sum_{i}\frac{I_{i}\times \Delta Y_{i}}{ln(\alpha_{i})}\times ln\left(\frac{Y^{0}}{Y^{t}}\right)$ induces the interpretation problem. It can indeed not be interpreted strictly as a structure effect, as it includes some part of the overall activity effect $ln(\frac{Y^{0}}{Y^{t}})$.

This problem decreases when the proportion of change becomes similar in all sectors:

$$\begin{array}{ccc} \alpha_{i} & \to & \alpha \\ Y^{t} & \to & \alpha \times Y^{0}\\ ln\left(\frac{Y^{0}}{Y^{t}}\right) & \to & -ln\left(\alpha \right)\\ \Delta E_{S} & \to & 0 \end{array}$$

This concludes the proof.

This problem is a significant concern, because the situation described in Claim 3 is most likely dominant in empirical studies.

In summary, the problem can be understood as follows. Calculating the effect of changing shares, as in the LMDI, is mathematically correct. However, except for the unlikely cases of a perfect switch (Claim 1) or a proportional change (Claim 2), it cannot be interpreted purely as a change in structure, because the change in the shares would also mean a change in overall activity. Reciprocally, a change in overall activity would lead to a change in the shares.^3^ Therefore, interpreting changes in shares as a structure effect can be misleading or even incorrect. In particular, changes in shares do not solely reflect changes between energy-efficient and energy-intensive sectors, which would often be the most natural interpretation of structure effects.

### An alternative to the LMDI: the Marshall-Edgeworth with structure effects decomposition (MESE)

To cope with the interpretation problem in conventional methods, we developed the following index decomposition approach, which by construction better reflects the logic of structure effects. This approach partly mirrors the idea of the (additive) Marshall-Edgeworth index [11] and includes the structure effect as described below. For an application, refer to Roux et al. [20]. An R package to implement this method can be found on GitHub (https://github.com/ncrx5/MEME.decomposition.git)

Unlike the LMDI approach, the MESE approach does not view structure effects as changes in shares. The MESE defines the effect of overall activity as changes in activity, assuming all sectors produce at the average energy intensity. The MESE defines structure effects as changes in activity weighted by the difference between the intensity in each sector and the average intensity.

#### Two possible decompositions of the change in energy use

Consider the following equation showing the drivers of an indicator of energy use embodied in the activity of a given sector *i*:

(2.2) $$E_{i}=Y_{i}\times I_{i}$$

where $E_{i}$ is the energy use from sector *I*, $Y_{i}\;$is the total output of sector *I* and $I_{i}\;$is the intensity (energy use per unit of product) of sector *i*. See appendix A2 for a decomposition with three drivers (e.g., sector size, energy requirement and emission intensity).

Taking the differential between time 0 and time *t* results in this:

(2.2a) $$E_{i}^{t}-E_{i}^{0}=Y_{i}^{t}\times I_{i}^{t}-Y_{i}^{0}\times I_{i}^{0}=Y_{i}^{t}\times I_{i}^{t}-Y_{i}^{0}\times I_{i}^{0}+Y_{i}^{0}\times I_{i}^{t}-Y_{i}^{0}\times I_{i}^{t}=\left(Y_{i}^{t}-Y_{i}^{0}\right)\times I_{i}^{t}+Y_{i}^{0}\times I_{i}^{t}-Y_{i}^{0}\times I_{i}^{0}=\left(Y_{i}^{t}-Y_{i}^{0}\right)\times I_{i}^{t}+\left(I_{i}^{t}-I_{i}^{0}\right)\times Y_{i}^{0}=\Delta Y_{i}\times I_{i}^{t}+\Delta I_{i}\times Y_{i}^{0}$$

Alternatively, $Y_{i}^{t}\times I_{i}^{0}$ instead of $Y_{i}^{0}\times I_{i}^{t}$ could have been added and subtracted in the second step, which would have led to:

(2.3) $$\begin{array}{ccc} E_{i}^{t}-E_{i}^{0} & = & \Delta Y_{i}\times I_{i}^{0}\\ & & +\Delta I_{i}\times Y_{i}^{t} \end{array}$$

#### Separating the structure effect from the overall activity effect

Set$\;\bar{I}=\frac{\sum_{i}}{\sum_{i}}$ the weighted average intensity of all sectors (i.e., the overall energy intensity in the country).

By adding and subtracting $\bar{I}$ in Eq. (2.2), the effect of a change in sector size can be split into a first part related to the average intensity (the effect of overall activity) and a second related to the difference between the intensity in each sector and the average intensity (the sector mix effect):

(2.4) $$E_{i}^{t}-E_{i}^{0}=\Delta Y_{i}\times \left(I_{i}^{t}-{\bar{I}}^{t}\right)+\Delta Y_{i}\times {\bar{I}}^{t}+\Delta I_{i}\times Y_{i}^{0}$$

Or from Eq. (2.3):

(2.5) $$E_{i}^{t}-E_{i}^{0}=\Delta Y_{i}\times \left(I_{i}^{0}-{\bar{I}}^{0}\right)+\Delta Y_{i}\times {\bar{I}}^{0}+\Delta I_{i}\times Y_{i}^{t}$$

Where:

$\Delta Y_{i}\times \left(I_{i}-\bar{I}\right)$ *= sector mix effect*

$\Delta Y_{i}\times \bar{I}$ *= overall activity effect*

$\Delta I_{i}\times Y_{i}$ *= intensity effect*

#### Total effect

The total effect is chosen as the arithmetic mean^4^ of each effect in Eqs. (2.4) and (2.5), and summed over all sectors.

Consequently, the sector mix effect is:

$$\Delta E_{mix}=\sum_{i}\frac{\begin{array}{c} \Delta Y_{i}\times \left(I_{i}^{t}-{\bar{I}}^{t}\right)+\Delta Y_{i}\times \left(I_{i}^{0}-{\bar{I}}^{0}\right) \end{array}}{2}$$

The effect of overall activity is:

$$\Delta E_{act}=\sum_{i}\frac{\Delta Y_{i}\times {\bar{I}}^{t}+\Delta Y_{i}\times {\bar{I}}^{0}}{2}$$

Finally, the intensity effect is:

$$\Delta E_{int}=\sum_{i}\frac{\Delta I_{i}\times Y_{i}^{0}+\Delta I_{i}\times Y_{i}^{t}}{2}$$

These equations can be used directly in applications.

In this MESE index decomposition, the overall activity effect is computed using the weighted mean of the intensity of all sectors. It therefore reflects the effect of a change in economic activity, assuming that this change occurred in the average sector (with a higher weight for larger sectors).

On the other hand, the sector mix effect reflects the change in the size of different sectors, weighted with the difference between the intensity of each sector and the weighted mean intensity of all sectors. Therefore, specialising in sectors with a higher than average energy intensity would increase the sector mix effect, and specialising in sectors with a lower than average energy intensity would decrease the sector mix effect.

In summary, in a MESE decomposition, the total sector mix effect will be positive (i.e., there will be an increase in energy consumption), but only if production increases in sectors with a higher than average intensity or decreases in sectors with a lower than average intensity. The opposite holds for a negative sector mix effect. If production increases in the average sector, or if it increases in the same proportion in all sectors, or if all sectors have the same intensity, the structure effect will be zero by construction. Therefore, the MESE is not subject to the interpretation problem described in Section 1 and better reflects the common understanding of the structure effect.

Refer to appendix A3 for a review of the situation with several mix effects (e.g., sector and fuel mix effect).

### Empirical example: energy carrier use in the USA

This section examines the extent to which the use of the MESE changes the decomposition results compared to the LMDI, focusing on energy carrier use in the USA.

#### Data for the empirical example

Using data from the multi-regional input-output database Exiobase 3 [21], we decomposed total energy carrier use in the USA from 1995 to 2016, comparing the LMDI 1 and the MESE. Exiobase version 3.6 integrates 44 countries and five rest-of-the-world regions and a variety of environmental, social and economic indicators and covers 163 industry sectors. These details make it the MRIO database with the highest and most consistent level of sector and product detail currently available [21,23]. Data on total energy carrier use in Exiobase 3 were constructed using statistics on energy consumption from the International Energy Agency (IEA). Despite the necessity of using monetary data in constant prices for decompositions over time [5], data on value added by each sector has also been taken from Exiobase 3 in current prices. No constant price version of Exiobase 3 is yet available. Despite the results of this empirical example not being open to further interpretation, our decision is justified as the prices do not influence the methodological comparison, which is our prior aim here. Furthermore, the data perfectly displays a consistent set of industries, which is widely used in academia, and our approach could therefore easily be checked using data from other countries.

#### Results of the empirical example

**Fig. 2:**
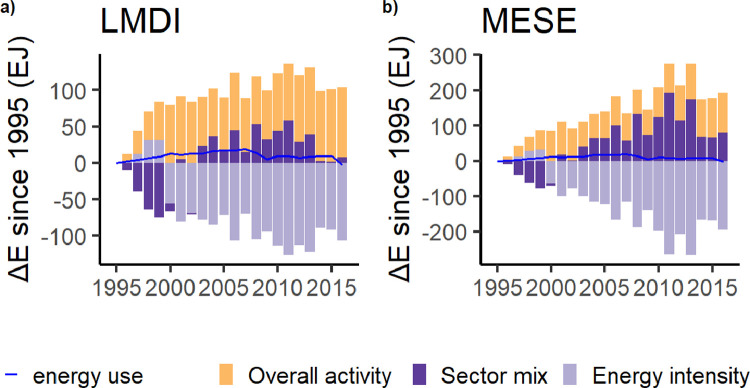
Decomposition of total energy carrier use in the USA for 163 sectors, using (a) the LMDI and b) the MESE.

It can be observed from the results of the empirical example that the MESE mainly changes the allocation between the sector mix and the overall activity effect, which is in line with our theoretical claims (Fig. 2). In this case, the MESE shows a larger effect on the sector mix (with some negative components of the overall activity effect being allocated to the sector mix effect). In the MESE decomposition, the effect of the sector mix was the largest upward-driving force of energy carrier use. Weber [22] found considerable structural change in the US economy around 2000 due to a quickly increasing trade deficit in manufacturing goods. Using the MESE decomposition makes this argument even more substantial and shows the importance of thoroughly analysing structure effects.

### Some desirable properties and limitations of the MESE decomposition

In addition to not being subject to the interpretation problem explained in Section 1, the MESE is a perfect decomposition method, meaning that it leaves no unexplained residuals. (The LMDI, other divisia indices, refined Laspeyres and modified Fischer ideal indices share this property.)

However, the MESE in its current form presents the following limitations:

1. The MESE is sensitive to small numbers, leading to missing or inconsistent data in the intensity denominator (in our case, sectoral output). This weakness may lead to sudden large energy intensities (especially if the spikes are in the base year). The problem even holds if the sectors of concern are small. In contrast to the LMDI, the problem does not hold only for zero values [7] but also for small non-zero values. When the energy intensity data in the base year seems inconsistent, one may consider keeping only the first development of the MESE (Eq. (2.4)). The problem can also be mitigated by filtering out observations with small values, generating a trade-off between consistency of results and losing data (see Appendix A4). This problem may lead to significant irregularities in the results and requires further improvements.
2. The MESE can be displayed with a fixed or rolling base year. Nevertheless, the results are dependent on the chosen base year. Adding up the results from a rolling base year (chaining) therefore leads to a cumulative error and should be avoided. Indeed, the contribution of a driver between t0 and t2 is not equal to the sum of the contributions between t0 and t1 plus t1 and t2. The idea is that each effect depends on the chosen base year so that the relative size of the effects will change with the base year, although the sum of all effects will always equal the absolute change in the impact (no residual). Refer to Appendix A5 for proof of this. This issue has also been stressed for other decomposition indexes, notably the Laspeyres and Paasche indexes, as well the AMDI and LMDI 1 [3,11].
3. Similar to refined Laspeyres and modified Fischer ideal indices, the length of the MESE equations increases with the number of considered factors (e.g., population, activity per capita, energy requirement, emission intensity). This trend may become inconvenient for a high number of factors, although the number of factors in most applications is somewhat limited.
4. Structure effects calculated by the MESE are dependent on the type of average used to calculate the average energy intensity. The weighted arithmetic mean intensity is a natural candidate as it represents the energy intensity of the overall production in a country, but other types of averages could also be used, which would affect the results.

## Conclusion

Determining the drivers behind energy use and related environmental impacts is essential for setting policy priorities. Index decomposition analysis reveals whether economic growth, sector changes, fuel mix changes or technological changes have the most significant effect on energy use and related emissions. Among the considered drivers, structure effects are of particular interest. Comparing the size of the activity and technology effects remains interesting, but the direction of these effects on energy use is usually straightforward: economic growth drives energy consumption upwards, whereas gains in energy efficiency reduce energy consumption per unit of output and thus dampen the increases resulting from economic growth. However, the effects of changes in the sector and fuel mix are more subtle and should therefore be analysed consistently.

The LMDI decomposition analysis presents elegant and enticing properties for discussing the drivers of energy consumption and associated environmental impacts. Consequently, it has become widely used in academia and public institutions’ environmental impact assessments. The method is often used to study structure effects.

Nonetheless, we have shown that the LMDI frequently yields counterintuitive results (such as a positive structure effect for changes between sectors of the same energy intensity). This limitation occurs because the LMDI uses the shares of that given mix (e.g., the share of a sector in the economy) to calculate the structure effect. Our viewpoint is that the LMDI does not reflect the intuitive interpretation of structure effects but is a rather abstract concept (“the effect of changing shares”), which is mathematically “correct” and yet difficult to interpret semantically. Changes in shares reflect a change in structure and overall activity. In particular, changes in shares cannot be interpreted as solely the effect of changes between energy-efficient and energy-intensive sectors. In our view, using the LMDI requires further research into what these “changes in shares” actually mean (especially for changes between sectors of the same intensity).

We suggested the MESE as an alternative decomposition method. The MESE does not define structure effects as changes in shares but as changes in activity weighted by the relative energy intensity of each sector. We believe this definition is closer to the intuitive and widely adopted interpretation of structure effects. Therefore, we recommend using the MESE when structure effects are included, especially if they are central to the analysis. However, all methods have pros and cons, and the MESE is subject to the limitations explained in Section 5 and possibly others we have not yet identified. Future research could improve our understanding of these pros and cons to determine for which purposes either method is a better fit.

Misinterpreting structure effects may generate significant issues, such as overestimating or underestimating the effect of economies specialising in particular sectors or using specific energy sources. For example, specialising in low energy sectors for services is often pursued as a strategy to reduce domestic energy use and CO_2_ emissions. Misestimating the effect of this specialization can lead to inappropriate policy recommendations. Similarly, misestimating the effect of replacing fossil fuels with carbon-free energy sources may discourage countries from engaging in green energy transitions.

## Declaration of Competing Interest

The authors declare that they have no known competing financial interests or personal relationships that could have appeared to influence the work reported in this paper.

## Footnotes

1 In this article, the terms *structure* and *mix effect* are used interchangeably.

2 However, the same argument would prevail for any other mix effect as the product mix, the energy mix or the mix of origin effect.

3 The shares and the overall activity are only independent in cases of a perfect switch (the change in the shares does not affect overall activity), or the proportional increase (the change in overall activity does not affect the shares).

4 One could also use a different type of mean as a geometric mean.
